# Seatbelt‐induced extrathoracic lung herniation

**DOI:** 10.1002/ccr3.3113

**Published:** 2020-07-16

**Authors:** Dimitrios Kitridis, Theodoros Karaiskos, Byron Chalidis, Nikiforos Galanis, Panagiotis Givissis

**Affiliations:** ^1^ 1st Orthopaedic Department George Papanikolaou Hospital, Aristotle University of Thessaloniki Thessaloniki Greece; ^2^ Cardiothoracic Surgery Department George Papanikolaou Hospital Thessaloniki Greece

**Keywords:** cardiothoracic surgery, emergency medicine, flail chest, orthopedics

## Abstract

Traumatic lung herniation is an uncommon complication of blunt chest trauma due to seatbelt injury. High index of suspicion, adherence to ATLS guidelines, and cooperation between different surgical specialties for the prompt stabilization of flail chest and primary or prosthetic closure of the defect may ensure a favorable outcome.

## Graphical abstract

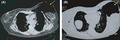

## 1 CASE PRESENTATION

**Figure 1:**
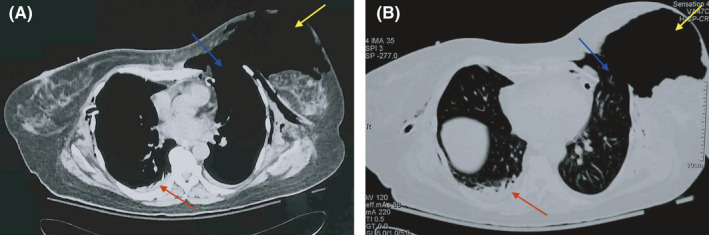
CT scan images of the patient's chest showing large left parasternal lung herniation (yellow arrow), costochondral disruptions (blue arrow), and a small left hemothorax (red arrow)

**Figure 2:**
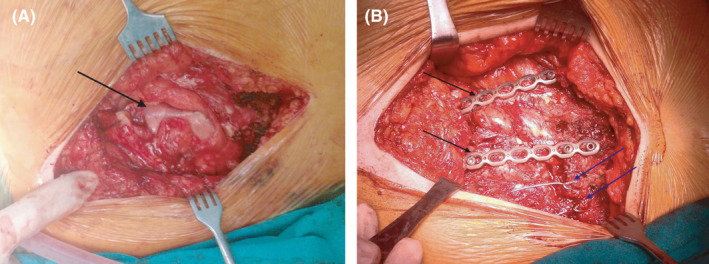
Intraoperative images showing the lung herniation (black arrow) under the subcutaneous fat (A), the reapproximation of the costal cartilages with wires (blue arrows) and the fixation of the ribs with reconstruction plates and screws (black arrows) (B)

**Figure 3:**
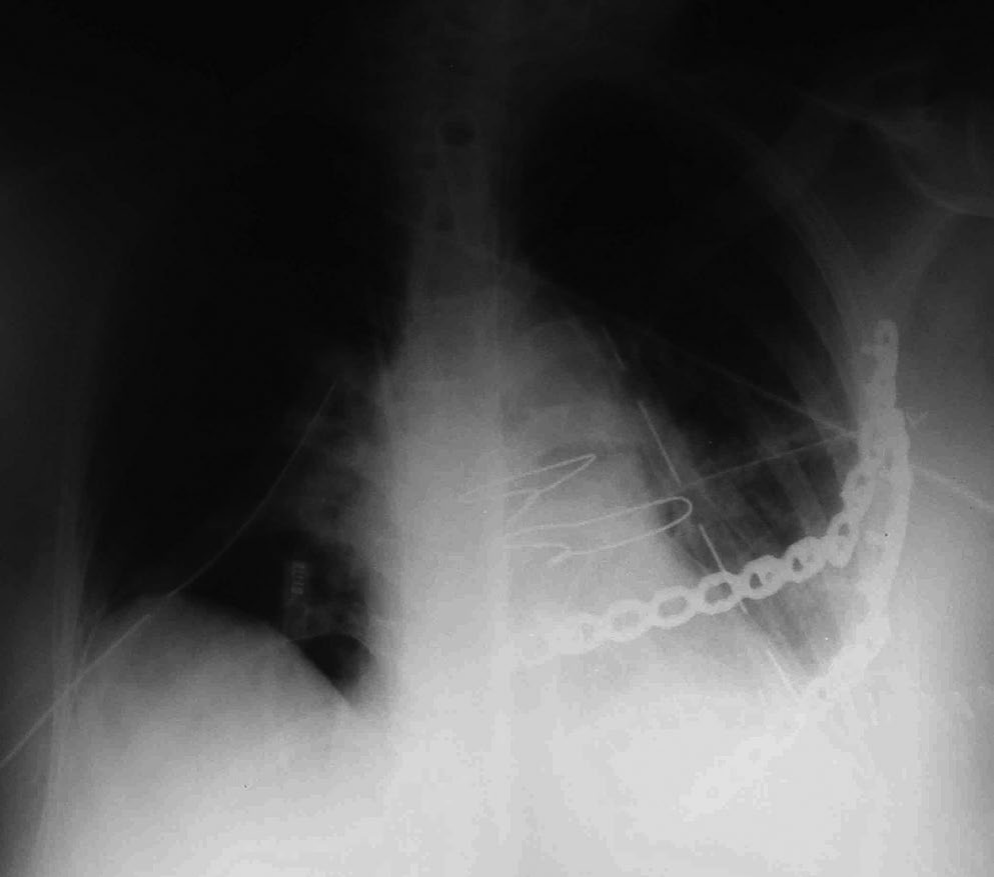
Postoperative radiograph showing the second and third costal cartilages approximated with sternal wires, and the fourth and fifth ribs fixed with reconstruction plates and screws

A 63‐year‐old woman involved in a road accident presented with blunt chest trauma and worsening respiratory distress. Her left pectoral region was deformed and hyper‐resonant, and paradoxical chest motion was noticed. The ATLS guidelines were followed, chest drains were placed bilaterally, and endotracheal intubation was performed. The CT revealed flail chest with a sternal body and multiple left‐sided rib fractures, sternocostal disruption of the upper ribs, a small hemothorax, and a large herniation of the left lung (Figure 1). She was taken to the theater later the same day and a midline skin incision extended to the left inframammary region was performed. The herniation was revealed under the subcutaneous fat, through the pectoralis major, which was then elevated from the chest wall (Video S1). The herniated lung was found to be viable. The second and third costal cartilages were reapproximated using sternal wires. The fourth and fifth ribs had segmental fractures and were fixed with reconstruction plates and screws (Figures 2 and 3). Any remaining defects were secured by a transosseous‐sutured polytetrafluoroethylene mesh. The chest drains were removed on the fifth postoperative day, and the patient was discharged on the seventh day; no activity restrictions were recommended.

## 2 DISCUSSION

Seatbelt use is associated with blunt chest trauma, and traumatic lung herniation is a rare complication.^1^ It usually occurs in the anterior thorax, where the intercostal muscle layer is thinnest.^2^ The management consists of ensuring stabilization of flail chest and primary or prosthetic closure of the defect.^1^

## CONFLICT OF INTEREST

There are no conflicts of interest associated with this publication, and there has been no financial support for this work that could have influenced its outcome.

## AUTHOR CONTRIBUTIONS

DK: drafted and submitted the manuscript. TK: obtained the photographs and contributed to patient care. BC: extracted patient history and assisted in completing edits. NG: critically reviewed the paper. PG: contributed to patient care and critically reviewed the manuscript.

## Supporting information

Video S1

## References

[1] Rice D , Bikkasani N , Espada R , Mattox K , Wall M . Seat belt‐related chondrosternal disruption with lung herniation. Ann Thorac Surg. 2002;73(6):1950‐1951. 12078797 10.1016/s0003-4975(01)03506-8

[2] Khalil MW , Masala N , Waller DA , Cardillo G . Surgical repair of post‐traumatic lung hernia using a video‐assisted open technique. Interact Cardiovasc Thorac Surg. 2008;7(3):506‐507. 18272541 10.1510/icvts.2007.168658

